# Orlistat for the treatment of antipsychotic-induced weight gain: an eight-week multicenter, randomized, placebo-controlled, double-blind trial

**DOI:** 10.1186/s12944-024-02214-w

**Published:** 2024-07-24

**Authors:** Peng Xie, Tiannan Shao, Yujun Long, Weiwei Xie, Yangjun Liu, Ye Yang, Yuyan Huang, Renrong Wu, Qijian Deng, Hui Tang

**Affiliations:** 1 Department of Psychiatry, National Clinical Research Center for Mental Disorders, National Center for Mental Disorders, and China National Technology Institute on Mental Disorders, The Second Xiangya Hospital of Central South University, 139# Renmin Middle RD, Changsha, 410011 Hunan China; 2 Department of Psychiatry, Second Affiliated Hospital, Zhejiang University School of Medicine, Hangzhou, 310009 Zhejiang China; 3 Department of Psychiatry, Affiliated Kangning Hospital of Ningbo University, Ningbo, 315201 Zhejiang China; 4 Department of Medical Epidemiology and Biostatistics, Karolinska Institutet, Stockholm, 17177 Sweden

**Keywords:** Antipsychotic, Orlistat, Weight gain, Serum lipid

## Abstract

**Background:**

Weight gain and metabolic disorders are commonly induced by antipsychotics. Orlistat is a lipase inhibitor used for weight control. The effect of orlistat on weight gain and metabolic disturbances in people (especially women) treated with antipsychotics has not been sufficiently studied. This study aimed to investigate the efficacy of orlistat in mitigating antipsychotic-induced weight gain and abnormal glycolipid metabolism.

**Methods:**

Patients with schizophrenia or bipolar disorder with a weight gain ≥ 7% after taking antipsychotics were recruited. Participants were randomly allocated to two groups: one received eight weeks of orlistat (360 mg/day) and the other received a placebo. Anthropometric and fasting serum biochemical parameters were measured at baseline, week 4 and week 8.

**Results:**

Sixty individuals (orlistat:placebo = 32:28) participated in the study. After controlling for the study center, the eight-week changes in body mass index (BMI), cholesterol (CHOL), high-density lipoprotein cholesterol (HDL-CH) and low-density lipoprotein cholesterol (LDL-CH) were significantly different between the groups. According to the mixed linear models, CHOL and LDL-CH were significantly lower in the orlistat group than in the control group at week 8. The week 0-to-8 slopes of BMI, CHOL and LDL-CH were also significantly lower in the orlistat group.

**Conclusions:**

These findings suggested that orlistat is an effective intervention for attenuating weight gain and serum lipid disturbances in antipsychotic-treated patients.

**Trial registration:**

ClinicalTrials.gov NCT03451734.

**Supplementary Information:**

The online version contains supplementary material available at 10.1186/s12944-024-02214-w.

## Background

Antipsychotics are widely used as an effective therapy for various mental disorders, including schizophrenia and affective disorders. However, antipsychotics may also induce metabolic abnormalities [1–4]. Huhn et al. analyzed data from 116 studies and found that 12 antipsychotics could induce significant weight gain in comparison with a placebo [5]. Subsequent meta-analyses indicated that blood lipids and glucose could also be influenced by multiple antipsychotics [6]. Without intervention, obesity and metabolic disorders may lead to cardiovascular disease [7], which shortens [8, 9] and impairs the quality of life and adherence to antipsychotics [10, 11]. According to previous literature, chlorpromazine, olanzapine and clozapine are at high risk for metabolic disturbance [12–14].

Antipsychotic-treated patients prefer high-fat food [15, 16]. Increased high-fat food cravings can predict clozapine-induced weight gain [17]. Furthermore, restrictions on fat intake contribute to attenuating antipsychotic-related weight gain [18, 19]. As the only clinically approved drug for obesity management in China [20], orlistat reduces systemic absorption of dietary fat by inhibiting gastric and pancreatic lipases in the gastrointestinal tract [21, 22]. Orlistat is not absorbable and is almost completely excreted in the feces [23, 24]. Previous studies have thoroughly investigated the effects of orlistat on obesity [25–28]. Long-term orlistat treatment reduces weight by 2–3 kg in obese people [26–29]. In addition, orlistat decreases fasting serum low-density lipoprotein cholesterol (LDL-CH), glucose and insulin levels, too [30–32]. For obese individuals with diabetes [33–36] and polycystic ovary syndrome [37–39], orlistat has also been shown to be capable of controlling weight and improving glycolipid metabolism.

Orlistat is also suitable for obese people with mental disorders because it does not influence plasma levels of psychotropic drugs [40]. However, there is limited research on the effectiveness of orlistat for treating antipsychotic-related obesity. Pavlovic reported a case, in which orlistat reduced the fasting blood glucose and weight of a man with schizophrenia on clozapine [41]. Joffe et al. administered orlistat to individuals taking olanzapine or clozapine for 16 weeks [42]. The results showed that orlistat effectively reduced weight in male participants, but not in female participants. Subsequent research confirmed the efficacy of orlistat in weight control for antipsychotic-treated men [43]. In fact, compared to men, women on antipsychotics have greater risks of weight gain, diabetes, and cardiovascular events [44]. Further evidence is needed to determine whether orlistat is beneficial to individuals, especially women, who take antipsychotics. This study was designed to assess the efficacy of orlistat in mitigating antipsychotic-induced weight gain. It was hypothesized that orlistat could control weight and improve glycolipid metabolism in antipsychotic-treated individuals.

## Methods

### Trial design

This clinical trial used a multicenter, randomized, double-blind, placebo-controlled design. Participants were assigned to the orlistat group and the control group at a 1:1 ratio. Interviews were conducted at three different time points: before treatment (week 0), four weeks after treatment began (week 4), and at the endpoint of the eight weeks of treatment (week 8).

Demographic information and medical history were collected at week 0. Height, Weight, hip circumference and waist circumference were measured at all three visits to calculate body mass index (BMI) and the waist-to-hip ratio (WHR). Fasting blood was collected at the three time points to analyze serum biochemical parameters including cholesterol (CHOL), high-density lipoprotein cholesterol (HDL-CH), LDL-CH, the HDL-CH–to–CHOL ratio (HD/CH), triglyceride (TG), glucose (GLU) and glycosylated hemoglobin (HbA1c). Participants were asked to report adverse events and the number of their remaining capsules at each visit as an indicator of treatment adherence. Taking fewer than two capsules per day was considered a treatment interruption.

This study is part of the project, “Optimizing and Individualizing the Pharmacological Treatment of First-episode Schizophrenic Patients” (ClinicalTrials.gov NCT03451734), which has been approved by the Medical Ethics Committee of the Second Xiangya Hospital of Central South University (ref: 2016S035) [45]. This study followed the Declaration of Helsinki, and the privacy rights of participants were always observed.

### Participants

Participants were recruited from two study centers: the Second Xiangya Hospital of Central South University (Changsha, Hunan) and the Affiliated Kangning Hospital of Ningbo University (Ningbo, Zhejiang). Individuals with schizophrenia or bipolar disorder were introduced to orlistat and the study protocol in detail. Only patients who were eligible for this study were recorded.

The inclusion criteria of this study are listed below: (a) aged between 16 and 60 years; (b) diagnosed with schizophrenia or bipolar disorder based on the Diagnostic and Statistical Manual of Mental Disorders, Fifth Edition (DSM-5); (c) receiving an antipsychotic at a constant dose for at least one month, with no plan to change the therapeutic regimen; (d) gaining weight after initiation of antipsychotic treatment and a current weight gain of ≥ 7% compared to the pre antipsychotic weight; and (e) providing written consent for themselves and their guardians.

Participants with the following conditions were excluded: (a) had a contraindication for orlistat, including an allergy and being overweight or obese due to physical disease; (b) were diagnosed with a major physical disease (including hyperthyroidism, hypothyroidism, hypertension, chronic liver disease, chronic kidney disease, immune system disorder and tumor); (c) were on a hypoglycemic drug, a hypolipemic drug, an anti-obesity drug, or any other therapy for weight loss (including systematic dietary control and high-intensity exercise) currently or during the past three months; (d) were pregnant or lactating; (e) had a history of psychoactive substance abuse or addiction; (f) were diagnosed with an eating disorder; and (g) were not taking medicine or interviewed as needed.

The study was monitored by an experienced psychiatrist. Participants’ mental status and adverse events were evaluated through interviews every four weeks. In addition, the research team was accessible to the participants and their guardians so that they could report any adverse events or changes in their conditions and consult with the researchers at any time. If participants experienced a health condition that required treatment violating the study protocol, researchers would help them access the necessary medical resources and exclude them from the study. Participants who met an exclusion criterion could still be interviewed if they were willing, and related data were analyzed by the intention-to-treat (ITT) method.

### Interventions

The orlistat group received orlistat capsules (0.12 g per capsule) and the control group received placebo capsules. The orlistat and placebo capsules were free of charge to participants. While continuing their original treatments, participants were required to take one capsule (orlistat or placebo) they received during or within one hour after each meal, three times a day for eight weeks.

### Outcomes

The primary outcomes were weight and BMI and the changes in them from baseline to the endpoint. To calculate BMI, weight (in kg) was divided by height (in m) squared. The secondary outcomes were levels of WHR, TG, CHOL, HDL-CH, LDL-CH, HD/CH, GLU and HbA1c, as well as the changes in these indicators from baseline to the endpoint.

### Sample size

Power and sample size calculations were conducted using the Power and Sample Size tool (http://powerandsamplesize.com/). The calculations were based on the results of a pre-experiment and designed to demonstrate an intergroup difference of 0.4 in changes in BMI from week 0 to week 8, with a power of 90% and a two-tailed significance level of 0.05. In addition, a standard deviation (SD) of 0.6 and an anticipated 20% loss to follow-up were also factored in. Eventually, it was determined that each treatment group would require a sample size of 26.

### Randomization and masking

Participants were assigned according to tables of random numbers generated by SPSS version 26 (IBM Corp, Armonk, NY, US). Orlistat and placebo capsules used in this trial were produced and packed by Hangzhou Zhongmeihuadong Pharmaceutical Co. The capsules looked the same and could only be distinguished from each other by production batch numbers. The lists recording the actual content of each batch were sealed in envelopes by the producer and were confidential to the researchers and participants during recruitment and visiting.

### Data analyses

Differences in variables over eight weeks were compared between groups by linear regression models. ITT analyses were achieved by filling in missing values via the last-observation-carried-forward (LOCF) method or by conducting mixed linear models. The study center was included in the linear regression models and mixed linear models as a covariate. In sensitivity analyses, taking a mood stabilizer was included as a covariate along with the study center. Stata version 17 (StataCorp LLC, College Station, TX, US) was used to construct mixed linear models. Other statistical analyses were conducted with SPSS version 26. The results were considered significant when *P* < 0.05 or 95% confidence intervals (CIs) did not include 0.

## Results

### Demographic data

Sixty eligible patients who used antipsychotics participated in this study from June 2019 to October 2021. Demographic and clinical information along with baseline outcomes are shown in Table 1. Briefly, 26 (43.3%) patients were diagnosed with schizophrenia and 34 (56.7%) were diagnosed with bipolar disorder. The illness duration was approximately 61.11 months (SD = 82.56). Thirty-eight (63.3%) participants were treated with olanzapine or clozapine, and none of the participants were treated with chlorpromazine. Other participants were on quetiapine, amisulpride, risperidone, lurasidone, aripiprazole or phenazopyridine (36.7%). Nineteen (31.7%) participants were taking two antipsychotics. The average age of the participants was 25.68 years (SD = 9.72). The participants were mainly (90.0%) females, and the overwhelming majority (93.3%) were Han Chinese. The demographic characteristics and the baseline outcomes were not significantly different between the groups (*P* > 0.05).

**Table 1:** Demographic information and baseline outcomes

Variable			Orlistat (*n* = 32)	Placebo (*n* = 28)	*χ*^2^, *df* or *U*	*P*
**Demographic Information**						
	Center					
		Second Xiangya Hospital	27 (84.4)	24 (85.7)	0.000, 1	> 0.999 ^a^
		Kangning Hospital	5 (15.6)	4 (14.3)		
	Sex					
		Male	4 (12.5)	2 (7.1)	0.067, 1	0.796 ^a^
		Female	28 (87.5)	26 (92.9)		
	Age					
			24.75 ± 8.44	26.75 ± 11.06	401	0.485
	Ethnic group					
		Han	32 (100.0)	24 (85.7)	2.871, 1	0.090 ^a^
		Others	0 (0.0)	4 (14.3)		
	Diagnosis					
		Schizophrenia	13 (40.6)	13 (46.4)	0.205, 1	0.651
		Bipolar disorder	19 (59.4)	15 (53.6)		
	Duration of disease (months)					
			58.06 ± 56.66	65.04 ± 108.57	300.5	0.225
	Taking olanzapine or clozapine					
		Yes	17 (65.6)	21 (60.7)	0.155, 1	0.694
		No	11 (34.4)	11 (39.3)		
	Taking two antipsychotics					
		Yes	9 (31.3)	10 (32.1)	0.006, 1	0.941
		No	19 (68.8)	22 (67.9)		
**Week 0 outcome**						
		BMI (kg/m^2^)	28.31 ± 4.35	27.12 ± 4.09	332	0.128
		WHR	0.90 ± 0.06	0.92 ± 0.07	265.5	0.262
		TG (mmol/L)	1.74 ± 1.14	1.87 ± 1.67	388	0.634
		CHOL (mmol/L)	4.60 ± 0.88	4.56 ± 0.95	378.5	0.533
		HDL-CH (mmol/L)	1.31 ± 0.33	1.24 ± 0.32	331	0.249
		LDL-CH (mmol/L)	2.77 ± 0.80	2.69 ± 0.82	345	0.353
		HD/CH	0.37 ± 0.27	0.31 ± 0.09	358	0.470
		GLU (mmol/L)	4.84 ± 1.14	5.00 ± 1.22	389	0.646
		HbA1c (%)	6.00 ± 1.96	5.43 ± 0.67	148.5	0.354

Numerical variables were presented as mean ± SD and compared by Mann-Whitney U test. Categorical variables were presented as N (%) and compared by chi-square test. ^a^ Continuity correction was applied where necessary. Abbreviations: BMI: Body Mass Index, WHR: Waist-to-Hip Ratio, TG: Triglyceride, CHOL: Cholesterol, HDL-CH: High-Density Lipoprotein Cholesterol, LDL-CH: Low-Density Lipoprotein Cholesterol, HD/CH: HDL-CH–to–CHOL ratio, GLU: Glucose, HbA1c: Glycosylated hemoglobin

**Fig. 1:**
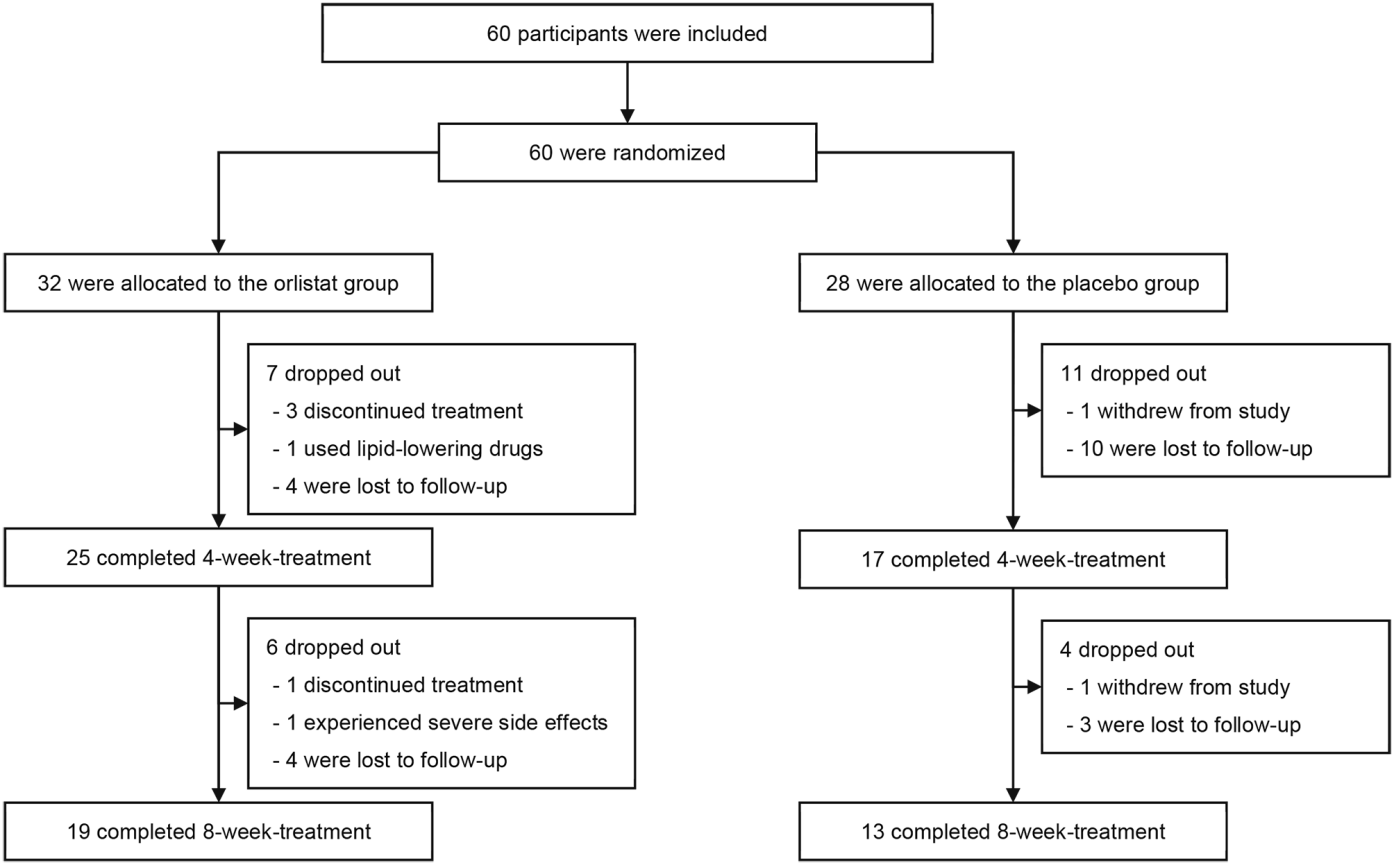
The flow chart of the study

As illustrated in Fig. 1, 32 participants were randomly assigned to the orlistat group and 28 were assigned to the placebo group. In the orlistat group, one patient did not take orlistat continuously for eight weeks but was followed up by week 4 and week 8; one patient started using atorvastatin after week 0 but was followed up by week 4 and week 8; and two patients stopped taking orlistat before week 4 but were followed up by week 4 and were lost by week 8. The data obtained from the visits mentioned above were included in the ITT analyses but were excluded when calculating differences in variables over eight weeks. Two patients in the placebo group were unwilling to further participate in this study after their last visit. Finally, 32 participants (53.3%) completed their 8-week treatments and all visits, including 19 (59.4%) from the orlistat group and 13 (46.4%) from the placebo group (*P* = 0.316).

### Comparisons of differences over eight weeks between groups

After eight weeks of treatments, weight decreased by 1.32 ± 2.16 kg and BMI decreased by 0.48 ± 0.77 kg/m^2^ in the orlistat group while weight increased by 0.47 ± 1.72 kg and BMI increased by 0.20 ± 0.65 kg/m^2^ in the placebo group. For the secondary outcomes, CHOL, HDL-CH, LDL-CH and GLU levels were also increased by placebo and reduced by orlistat treatment. After controlling for the study center in linear regression models, 8-week changes in weight, BMI, CHOL, LDL-CH and HDL-CH significantly differed between the two groups (*b* = -1.77 to -0.2, upper bounds of 95% CIs < 0, Table 2). Linear regression models were performed again after filling in missing values by the LOCF method, and the results remained almost the same (Table S1).

**Table 2:** Comparisons of 8-week changes in metabolic parameters between the groups

Variable	Difference between week 8 and week 0		Linear Regression (*n* = 32)			
	Orlistat (*n* = 19)	Placebo (*n* = 13)				
			*b*	SE	*P*	95% CI
Weight (kg)	-1.32 ± 2.16	0.47 ± 1.72	-1.77	0.72	0.021	[-3.25, -0.29]
BMI (kg/m^2^)	-0.48 ± 0.77	0.2 ± 0.65	-0.68	0.26	0.015	[-1.21, -0.14]
WHR	0.02 ± 0.03	0 ± 0.03	0.02	0.01	0.15	[-0.01, 0.04]
TG (mmol/L)	-0.02 ± 0.99	-0.45 ± 0.54	0.42	0.32	0.197	[-0.24, 1.08]
CHOL (mmol/L)	-0.5 ± 0.55	0.14 ± 0.40	-0.62	0.17	0.001	[-0.97, -0.26]
HDL-CH (mmol/L)	-0.16 ± 0.30	0.04 ± 0.11	-0.2	0.09	0.035	[-0.39, -0.02]
LDL-CH (mmol/L)	-0.33 ± 0.45	0.21 ± 0.28	-0.53	0.14	0.001	[-0.82, -0.24]
HD/CH	0.01 ± 0.09	-0.01 ± 0.03	0.02	0.03	0.572	[-0.04, 0.07]
GLU (mmol/L)	-0.24 ± 0.93	0.55 ± 1.26	-0.79	0.42	0.071	[-1.66, 0.07]
HbA1c (%)	-0.05 ± 0.28	-0.03 ± 0.20	-0.04	0.12	0.718	[-0.29, 0.20]

Data were presented as mean ± SD. Participants who violated the study protocol were excluded. *Abbreviations*: BMI: Body Mass Iindex, WHR: Waist-To-Hip Ratio, TG: Triglyceride, CHOL: Cholesterol, HDL-CH: High-Density Lipoprotein Cholesterol, LDL-CH: Low-Density Lipoprotein Cholesterol, HD/CH: HDL-CH–to–CHOL ratio, GLU: glucose, HbA1c: glycosylated hemoglobin, CI: Confidence Interval

### ITT analyses

**Fig. 2:**
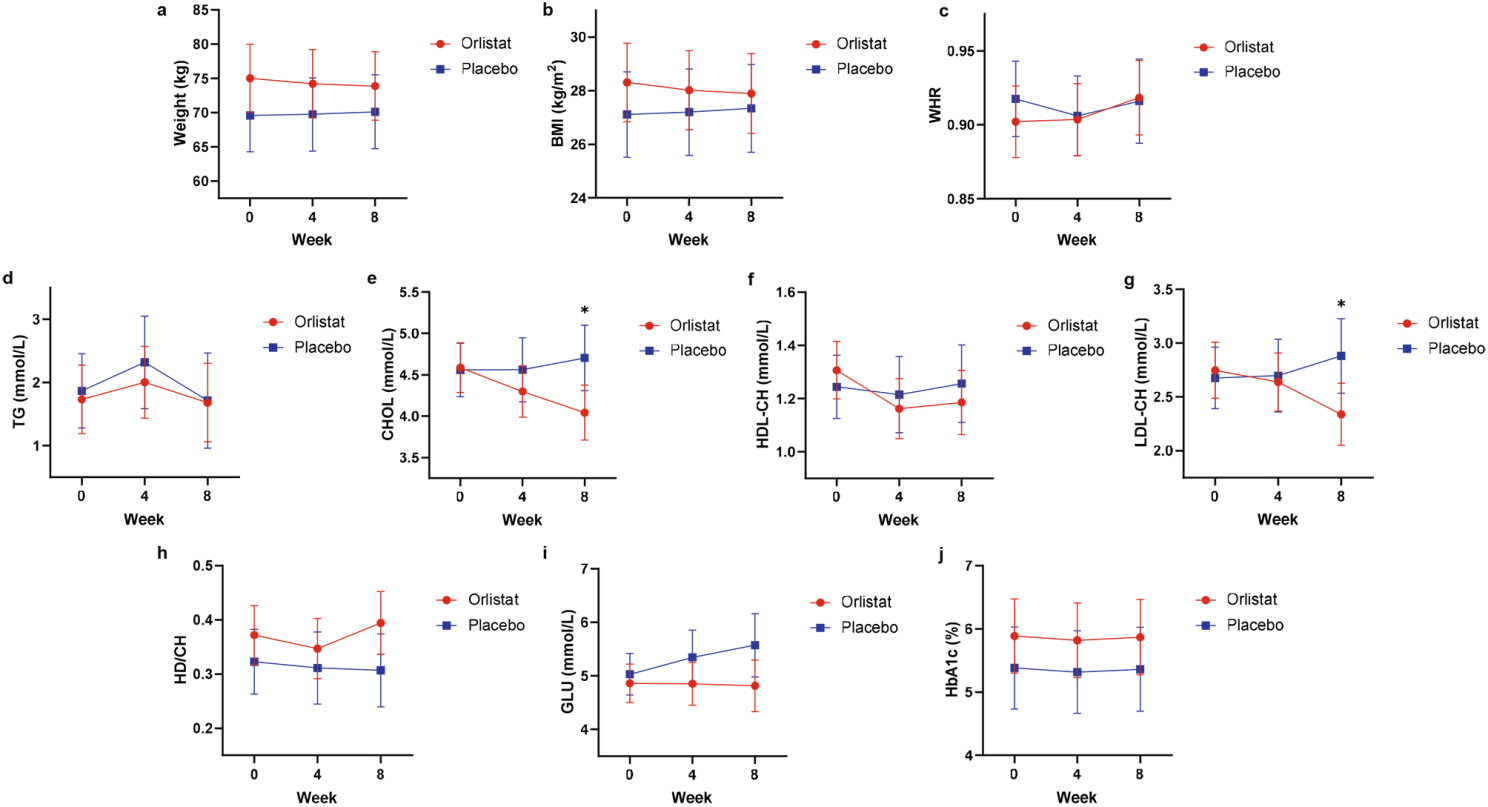
Mixed linear models for comparisons of metabolic parameters. Data from all visits were analyzed using the intent-to-treat method. (**a**–**j**) show results for weight, BMI, WHR, TG, CHOL, HDL-CH, LDL-CH, HD/CH, GLU and HbA1c, respectively. The dots and error bars represent margins and 95% confidence intervals. ✱ Significant difference between groups (95% confidence interval for the contrast between margins does not include 0). Abbreviations: BMI: body mass index, WHR: waist-to-hip ratio, TG: triglyceride, CHOL: cholesterol, HDL-CH: high-density lipoprotein cholesterol, LDL-CH: low-density lipoprotein cholesterol, HD/CH: HDL-CH–to–CHOL ratio, GLU: glucose, HbA1c: glycosylated hemoglobin

Considering the high proportion of participants who dropped out, ITT analyses were conducted to further verify the effects of orlistat on metabolism-related indices. As shown in Fig. 2, the margin of BMI increased from 27.11 (95% CI: [25.51, 28.71]) to 27.34 (95% CI: [25.70, 28.98]) kg/m^2^ in the placebo group (slope: 0.23, 95% CI: [-0.20, 0.66]) and decreased from 28.31 (95% CI: [26.84, 29.77]) to 27.89 (95% CI: [26.40, 29.38]) kg/m^2^ in the orlistat group (slope: -0.41, 95% CI: [-0.75, -0.08]). Similarly, the margins of CHOL, LDL-CH and GLU also decreased in the orlistat group and increased in the placebo group. No variables significantly differed between the groups at week 0 and week 4. However, at week 8, the CHOL (contrast = -0.66, 95% CI: [-1.18, -0.15]) and LDL-CH (contrast = -0.54, 95% CI: [-0.99, -0.09]) levels in the orlistat group were significantly lower in comparison with those in the placebo group. The week-0-to-8 slopes of BMI (contrast = -0.64, 95% CI: [-1.19, -0.10]), CHOL (contrast = -0.69, 95% CI: [-1.09, -0.28]) and LDL-CH (contrast = -0.61, 95% CI: [-0.97, -0.26]) were significantly lower in the group treated with orlistat. ITT analyses were also repeated without data from the invalid visits mentioned in § 3.1, and similar results were obtained (Figure S1).

### Subgroup analyses

#### Sex

Previous studies have indicated that orlistat is not effective for females [42, 43]. Therefore, the metabolic parameters of the female participants were analyzed again (Figure S2). The outcomes were similar to the previous outcomes. CHOL and LDL-CH levels did not differ between groups at baseline but were lower in the orlistat group after eight weeks (CHOL: contrast = -0.75, 95% CI: [-1.26, -0.24]; LDL-CH: contrast = -0.68, 95% CI: [-1.13, -0.23]). Weight, BMI, CHOL, LDL-CH, HDL-CH and GLU increased in the placebo group but decreased in the orlistat group. The differences in the week-0-to-8 slopes between the groups were significant for weight (contrast = -1.55, 95% CI: [-3.01, -0.09]), BMI (contrast = -0.61, 95% CI: [-1.16, -0.06]), CHOL (contrast = -0.72, 95% CI: [-1.15, -0.28]), HDL-CH (contrast = -0.20, 95% CI: [-0.34, -0.07]) and LDL-CH (contrast = -0.62, 95% CI: [-1.00, -0.24]). The number of males was too low so ITT analyses were not repeated for male participants.

#### Diagnosis

In participants with schizophrenia, orlistat was ineffective to weight or BMI, but the slopes of CHOL (contrast = -0.93, 95% CI: [-1.56, -0.30]) and LDL-CH (contrast = -0.87, 95% CI: [-1.37, -0.36]) were still lower in the orlistat group. In participants with bipolar disorder, the slopes of weight (contrast = -1.32, 95% CI: [-4.90, -0.00]) and BMI (contrast = -0.94, 95% CI: [-1.83, -0.05]) were significantly lower for those treated with orlistat; CHOL did not differ between groups at week 0 but the orlistat group had a significantly lower CHOL level at week 8 (contrast = -0.79, 95% CI: [-1.54, -0.03]) (Figure S3).

#### Metabolic risk levels of antipsychotics

In participants taking olanzapine or clozapine, the slopes of weight (contrast = -2.55, 95% CI: [ -4.36, -0.75]), BMI (contrast = -0.97, 95% CI: [-1.63, -0.31]), CHOL (contrast = -0.74, 95% CI: [-1.20, -0.28]) and LDL-CH (contrast = -0.58, 95% CI: [-1.01, -0.14]) were significantly reduced by orlistat. In participants treated with other antipsychotics, week-0-to-8 slopes were not different between groups; CHOL (contrast = -1.17, 95% CI: [-2.07, -0.27]), LDL-CH (contrast = -0.83, 95% CI: [-1.55, -0.12]) and GLU (contrast = -1.20, [-2.28, -0.12]) were significantly lower in the orlistat group after interventions, while no indicators differed between the groups at baseline (Figure S4).

#### Numbers of antipsychotics

For participants treated with one antipsychotic, the week-0-to-8 slopes of BMI (contrast = -0.76, 95% CI: [-1.48, -0.04]), CHOL (contrast = -0.86, 95% CI: [-1.34, -0.37]) and LDL-CH (contrast = -0.71, 95% CI: [-1.14, -0.27]) in the orlistat group were significantly lower; CHOL and LDL-CH were comparable for the two groups at baseline but were significantly lower in the orlistat group at week 8 (CHOL: contrast = -0.65, 95% CI: [-1.29, -0.02]; LDL-CH: contrast = -0.53, 95% CI: [-1.05, -0.00]). In participants treated with two antipsychotics, metabolic parameters did not differ between groups at any time point; slopes were not different between groups (Figure S5).

### Sensitivity analyses

Twenty-five (41.7%) participants were taking lithium, valproate, or lamotrigine. The effect of mood stabilizers on body weight is controversial and was therefore included in sensitivity analyses [46]. Week-0-to-8 slopes of weight (contrast = -1.66, 95% CI: [-3.15, -0.18]), BMI (contrast = -0.64, 95% CI: [-1.19, -0.10]), CHOL (contrast = -0.69, 95% CI: [-1.09, -0.29]) and LDL-CH (contrast = -0.61, 95% CI: [-0.97, -0.26]) were significantly different between groups. CHOL (contrast = -0.63, 95% CI: [-1.17, -0.10]) and LDL-CH (contrast = -0.52, 95% CI: [-0.99, -0.05]) were significantly lower in the orlistat group after the intervention while no variable was significantly different between the groups at baseline (Figure S6).

### Adverse events

Sixteen participants (50.0%) in the orlistat group and two (7.1%) in the placebo group reported newly-emerged diarrhea, oily feces, or oil leakage from the anus during the study (*P* < 0.001). No other severe side effects related to orlistat were reported.

## Discussion

This study is a randomized, placebo-controlled, and double-blind clinical trial focusing on the efficiency of orlistat on metabolic side effects caused by antipsychotics. The results indicate that orlistat contributes to weight control and blood lipid homeostasis in antipsychotic-treated people, which is consistent with previous studies conducted in obese people [25–31, 47, 48]. However, the effects of orlistat on glucose metabolism are not credible, which is inconsistent with previous reports [29, 31, 35, 48–50]. The level of HbA1c is relatively stable, so an 8-week intervention with orlistat may be too short to influence its level. For fasting serum glucose, the mixed linear model tended to decrease after orlistat treatment, but the difference was not statistically significant; this may be explained by the dispersion of the data. Further research should be carried out with larger sample sizes to provide confirmative evidence that orlistat repairs antipsychotic-related glucose metabolic disturbances.

Few studies have examined the efficiency of orlistat on antipsychotic-related weight gain. Previous articles reported that in olanzapine- or clozapine-treated people, orlistat was effective in weight control for men but not women [42, 43]. However, among individuals taking antipsychotics, women are at a greater risk of metabolic syndrome than men are [51–54]. Women are also more interested in becoming slimmer [55, 56], which makes the effectiveness of orlistat even more meaningful for them. Although the majority of the samples were from females, protective effects of orlistat on metabolism were still observed, which persisted when only females were analyzed. According to previous articles, orlistat can also decrease body weight and serum lipids and improve insulin sensitivity in obese females diagnosed with polycystic ovary syndrome [37, 39, 57]. Overall, orlistat may still be beneficial for females who are overweight or obese due to taking antipsychotics.

Metabolic syndrome is equally prevalent among patients with bipolar disorder and patients with schizophrenia [58, 59]. However, it is not clear whether the prognosis of antipsychotic-related metabolic abnormalities differs between schizophrenia and bipolar disorder [60–62]. Furthermore, the use of olanzapine and clozapine results in a greater risk for metabolic disturbance than other antipsychotics [13, 58, 62]. This study presents an exploration of the effects of diagnosis and treatment regimen on the efficacy of orlistat by subgroup analyses. The results revealed that orlistat was able to control weight and serum lipids in antipsychotic-treated participants with schizophrenia and bipolar disorder. Orlistat also effectively decreased the weight gain and increased serum lipids induced by antipsychotics with high metabolic risk, and increased serum lipids and glucose levels induced by antipsychotics with medium and low metabolic risk. In addition, orlistat was ineffective for participants who took two antipsychotics. However, considering the sample size, the results of subgroup analyses may not be representative. Mood stabilizers may also lead to weight gain [63]. Compared to antipsychotics, mood stabilizers have less of an effect on weight [64–66]. It has also been argued that there is insufficient evidence to prove that mood stabilizers (such as lithium, valproate and lamotrigine) can significantly increase weight [46, 67, 68]. Sensitivity analyses that included the presence or absence of mood stabilizers as a covariate were conducted in mixed linear models. The results still showed that orlistat alleviated antipsychotic-induced weight gain and dyslipidemia.

Eating behaviors are related to the efficiency of orlistat [69]. Orlistat treatment is usually combined with a low-fat diet to fully exert its effects [30–32, 38, 39, 70–72]. However, low compliance is common in weight loss programs [73]. People with negative or depressive symptoms may lack the motivation to follow a weight-control diet, especially when their appetite is elevated by antipsychotics. In this study, there was no requirement for physical exercise or diet, which may have limited the efficiency of orlistat but allowed the study to reflect the real situation. Taghizadeh et al. also observed a decrease in body weight in a group of overweight people who received orlistat treatment while maintaining their lifestyles [74]. In two other large studies, orlistat remained capable of controlling body weight after switching from a hypocaloric diet to a weight-maintenance diet [30, 31]. For people who cannot change their lifestyle, orlistat alone is still helpful for weight control.

Orlistat is generally safe because its absorption and accumulation are negligible [24, 31]. In this study, orlistat rarely induced side effects other than gastrointestinal adverse events, which is consistent with previous findings [30–32, 70, 75]. Even gastrointestinal events tend to occur early in orlistat treatment and may resolve spontaneously [30, 31, 76]. In addition, orlistat does not influence plasma concentrations of psychotropic drugs [40]. While benefitting body weight and lipid metabolism, orlistat does not interfere with antipsychotic treatment [77]. Therefore, orlistat is suitable for overweight or obese people taking antipsychotics.

### Study strengths and limitations

The efficacy of orlistat for antipsychotic metabolic side effects has not been adequately studied. Previous articles reported that orlistat was able to alleviate olanzapine- and clozapine-induced weight gain and abnormal serum lipid and glucose levels, but was only effective in men with schizophrenia [41–43]. In contrast, this study proved that orlistat is effective in antipsychotic-treated women and patients with bipolar disorder. The present study also confirmed that orlistat is effective for treating metabolic abnormalities caused by antipsychotics other than olanzapine and clozapine. Furthermore, the study revealed that orlistat is effective only in patients treated with a single antipsychotic. In summary, this study fills the gap of previous studies and affirms the efficacy of orlistat in managing the metabolic side effects induced by antipsychotics.

This study has a few limitations. First, the sample size was small. During the coronavirus disease (COVID-19) pandemic, recruitment was suspended for months because of pandemic prevention and control policies and the delayed supply of study drugs. Further, more than 1/3 of the participants dropped out, which is similar to the findings of some long-term studies on orlistat [30, 31, 40, 78]. Some participants refused to leave home for follow-up visits to avoid the infection of severe acute respiratory syndrome coronavirus 2 (SARS-CoV-2). Meanwhile, the characteristic side effects of orlistat caused difficulties in blinding. Many participants were convinced that they were taking a placebo due to the absence of weight loss and steatorrhea and were unwilling to continue the study. As a result, more individuals completed the study in the orlistat group than in the placebo group, which was consistent with the higher incidence of gastrointestinal adverse events in the orlistat group. In previous studies with large samples, the drop-out ratios in placebo groups were also greater [30, 31, 36]. A placebo lead-in period may help screen out participants with low adherence, thus reducing attrition [79]. However, this was a short-term study, and the availability of study drugs was limited, so there was no lead-in period. The sample size also limited the quality of subgroup analyses. In summary, the effects of orlistat on antipsychotic-induced metabolic disorders should be validated in larger samples exclusive to individuals with poor compliance.

## Conclusions

In antipsychotic-treated patients, eight weeks of orlistat treatment can control body weight and improve fasting lipid levels. Orlistat is also effective in antipsychotic-treated women. Patients with bipolar disorder and patients with schizophrenia can both benefit from orlistat treatment. In addition, orlistat is effective at alleviating metabolic disturbances induced by antipsychotics, regardless of metabolic risk level. However, orlistat is not effective in patients taking multiple antipsychotics.

### Electronic supplementary material

Below is the link to the electronic supplementary material.

Supplementary Material 1

## Data Availability

Data are accessible through corresponding authors upon reasonable request.

## Abbreviations

BMI: Body Mass Index
WHR: Waist-to-Hip Ratio
TG: Triglyceride
CHOL: Cholesterol
HDL-CH: High-Density Lipoprotein Cholesterol
LDL-CH: Low-Density Lipoprotein Cholesterol
HD/CH: HDL-CH–to–CHOL ratio
GLU: Glucose
HbA1c: glycosylated Hemoglobin
ITT: Intention-To-Treat
SD: Standard Deviation
CI: Confidence Interval
LOCF: Last-Observation-Carried-Forward
