# Supplementary material for: Orlistat for the treatment of antipsychotic-induced weight gain: an eight-week multicenter, randomized, placebo-controlled, double-blind trial
Source: Lipids Health Dis. 2024 Jul 24;23:225. doi: 10.1186/s12944-024-02214-w (PMC11267745; doi:10.1186/s12944-024-02214-w)
Supplement: Supplementary file 1 — Supplementary Material 1 [file 12944_2024_2214_MOESM1_ESM.pdf]

Orlistat for the treatment of antipsychotic-induced weight gain: An eight-week multicenter, randomized, placebo-controlled, double-blind trial

Peng Xie, Tiannan Shao, Yujun Long, Weiwei Xie, Yangjun Liu, Ye Yang, Yuyan Huang, Renrong Wu, Qijian Deng, Hui Tang

Corresponding authors:
Qijian Deng: dengqijian@csu.edu.cn
Hui Tang: tanghui2017@csu.edu.cn
Department of Psychiatry, National Clinical Research Center for Mental Disorders, and National Center for Mental Disorders, the Second Xiangya Hospital of Central South University, 139# Renmin Middle RD, Changsha 410011, Hunan, China.

Table S1. Comparisons of 8-week changes of metabolic parameters between the groups after filling in missing values

Variable	Difference between week 8 and week 0		Linear Regression (n = 60)			
	Orlistat (n = 32)	Placebo (n = 28)	b	SE	P	95% CI
Weight (kg)	-0.94 ±2.25	0.28 ±1.32	-1.21	0.48	0.015	[-2.18, -0.24]
BMI (kg/m^2)	-0.35 ±0.81	0.13 ±0.53	-0.47	0.18	0.012	[-0.84, -0.11]
WHR	0.01 ±0.03	0.00 ±0.03	-0.01	0.01	0.671	[-0.03, 0.02]
TG (mmol/L)	0.06 ±0.76	0.13 ±1.70	-0.08	0.34	0.824	[-0.76, 0.61]
CHOL (mmol/L)	-0.37 ±0.67	0.12 ±0.31	-0.48	0.14	0.001	[-0.75, -0.21]
HDL-CH (mmol/L)	-0.10 ±0.26	0.00 ±0.10	-0.10	0.05	0.058	[-0.21, 0.00]
LDL-CH (mmol/L)	-0.26 ±0.54	0.12 ±0.26	-0.38	0.11	0.002	[-0.61, -0.15]
HD/CH	0.01 ±0.08	-0.01 ±0.03	0.02	0.02	0.235	[-0.01, 0.05]
GLU (mmol/L)	0.01 ±0.75	0.24 ±0.87	-0.24	0.21	0.271	[-0.67, 0.19]
HbA1c (%)	-0.04 ±0.22	-0.02 ±0.13	-0.02	0.06	0.785	[-0.14, 0.10]

Data were presented as mean±SD. All participants were included. Missing data were processed by the last-observation-carried-forward method. Abbreviations: BMI: body mass index, WHR: waist-to-hip ratio, TG: triglyceride, CHOL: cholesterol, HDL-CH: high-density lipoprotein cholesterol, LDL-CH: low-density lipoprotein cholesterol, HD/CH: HDL-CH–to–CHOL ratio, GLU: glucose, HbA1c: glycosylated hemoglobin, CI: confidence interval.

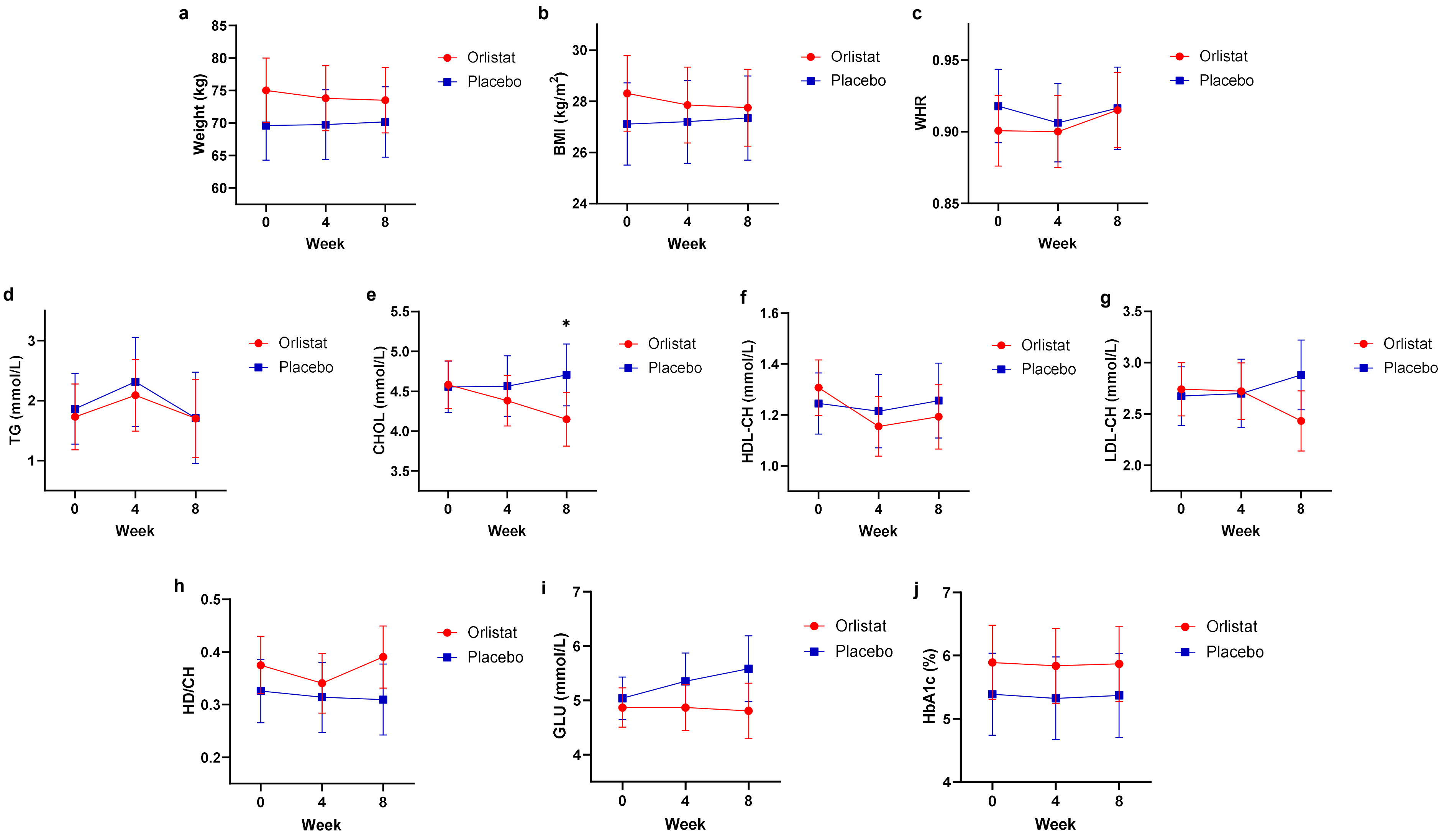

Figure S1. Mixed linear models for comparisons of metabolic parameters

Data were analyzed using the intent-to-treat method. Invalid visits (defined in § 3.1) were excluded. (a) to (j) show results for weight, BMI, WHR, TG, CHOL, HDL-CH, LDL-CH, HD/CH, GLU and HbA1c, respectively. The dots and error bars represent margins and 95% confidence intervals. ✱ Significant difference between groups (95% confidence interval for the contrast between margins does not include 0). Abbreviations: BMI: body mass index, WHR: waist-to-hip ratio, TG: triglyceride, CHOL: cholesterol, HDL-CH: high-density lipoprotein cholesterol, LDL-CH: low-density lipoprotein cholesterol, HD/CH: HDL-CH–to–CHOL ratio, GLU: glucose, HbA1c: glycosylated hemoglobin.

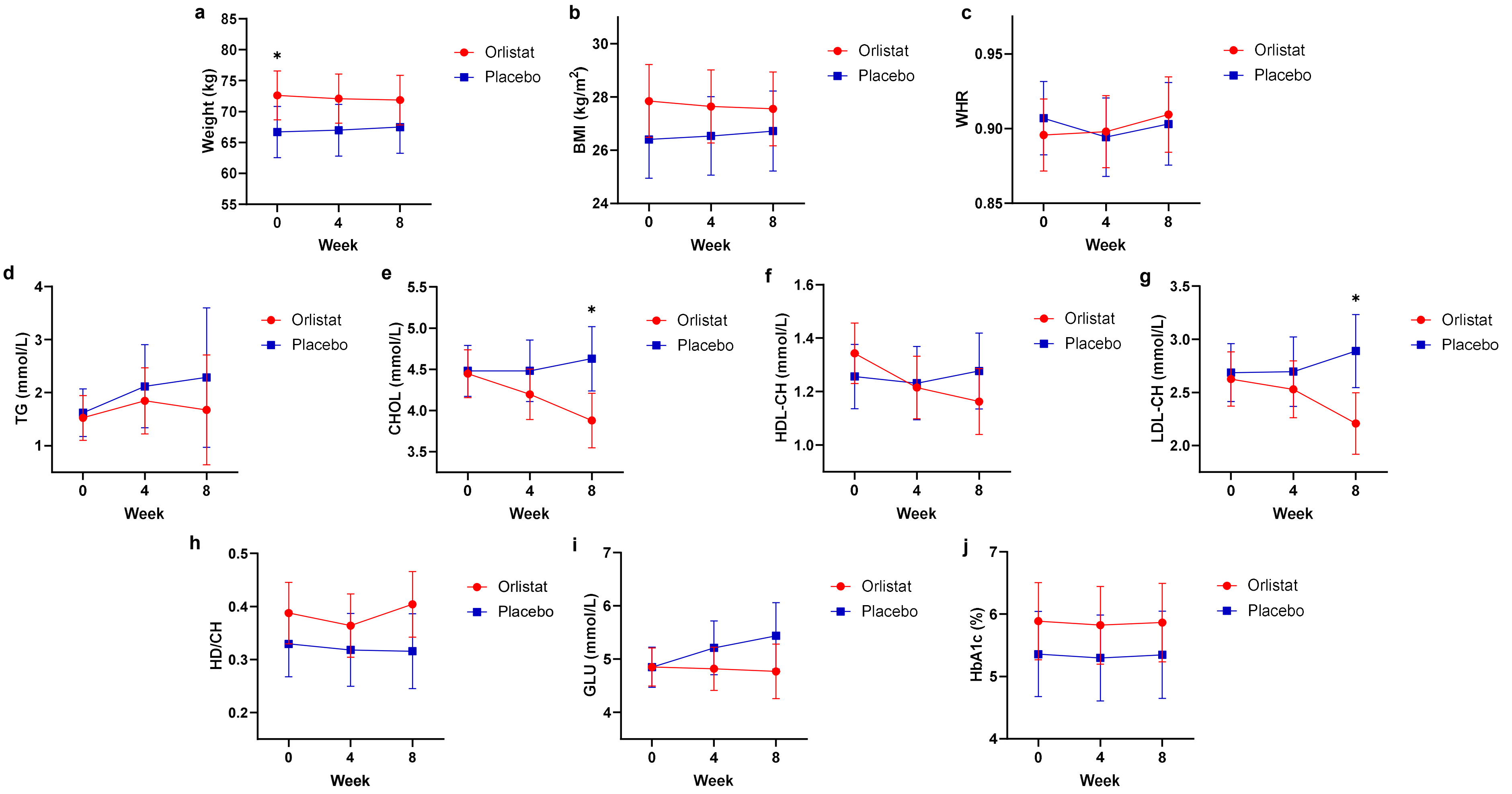

Figure S2. Mixed linear models analyzing metabolic parameters of female participants

Data from female participants ($n_{\text{orlistat}} = 28$, $n_{\text{placebo}} = 26$) were analyzed using the intent-to-treat method. Males were too few and thus not analyzed. (a) to (j) show results for weight, BMI, WHR, TG, CHOL, HDL-CH, LDL-CH, HD/CH, GLU and HbA1c, respectively. The dots and error bars represent margins and 95% confidence intervals. ✱ Significant difference between groups (95% confidence interval for the contrast between margins does not include 0). Abbreviations: BMI: body mass index, WHR: waist-to-hip ratio, TG: triglyceride, CHOL: cholesterol, HDL-CH: high-density lipoprotein cholesterol, LDL-CH: low-density lipoprotein cholesterol, HD/CH: HDL-CH–to–CHOL ratio, GLU: glucose, HbA1c: glycosylated hemoglobin.

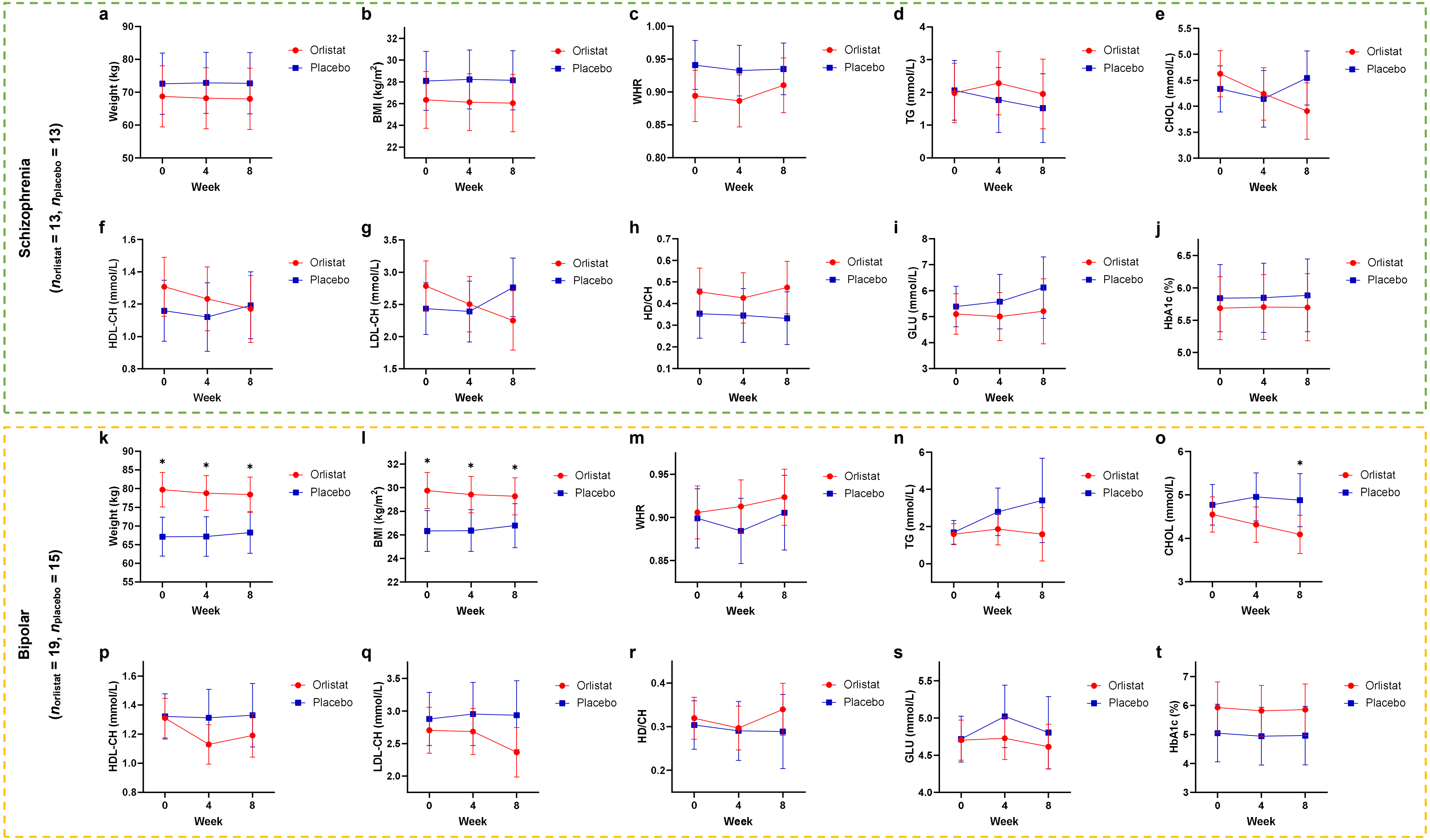

Figure S3. Mixed linear models: subgroup analyses by diagnosis

Data were split according to diagnosis and were analyzed using the intent-to-treat method. (a) to (j) show results for weight, BMI, WHR, TG, CHOL, HDL-CH, LDL-CH, HD/CH, GLU and HbA1c in patients with schizophrenia. (k) to (t) show results for weight, BMI, WHR, TG, CHOL, HDL-CH, LDL-CH, HD/CH, GLU and HbA1c in patients with bipolar disorder. The dots and error bars represent margins and 95% confidence intervals. ✱ Significant difference between groups (95% confidence interval for the contrast between margins does not include 0). Abbreviations: BMI: body mass index, WHR: waist-to-hip ratio, TG: triglyceride, CHOL: cholesterol, HDL-CH: high-density lipoprotein cholesterol, LDL-CH: low-density lipoprotein cholesterol, HD/CH: HDL-CH–to–CHOL ratio, GLU: glucose, HbA1c: glycosylated hemoglobin.

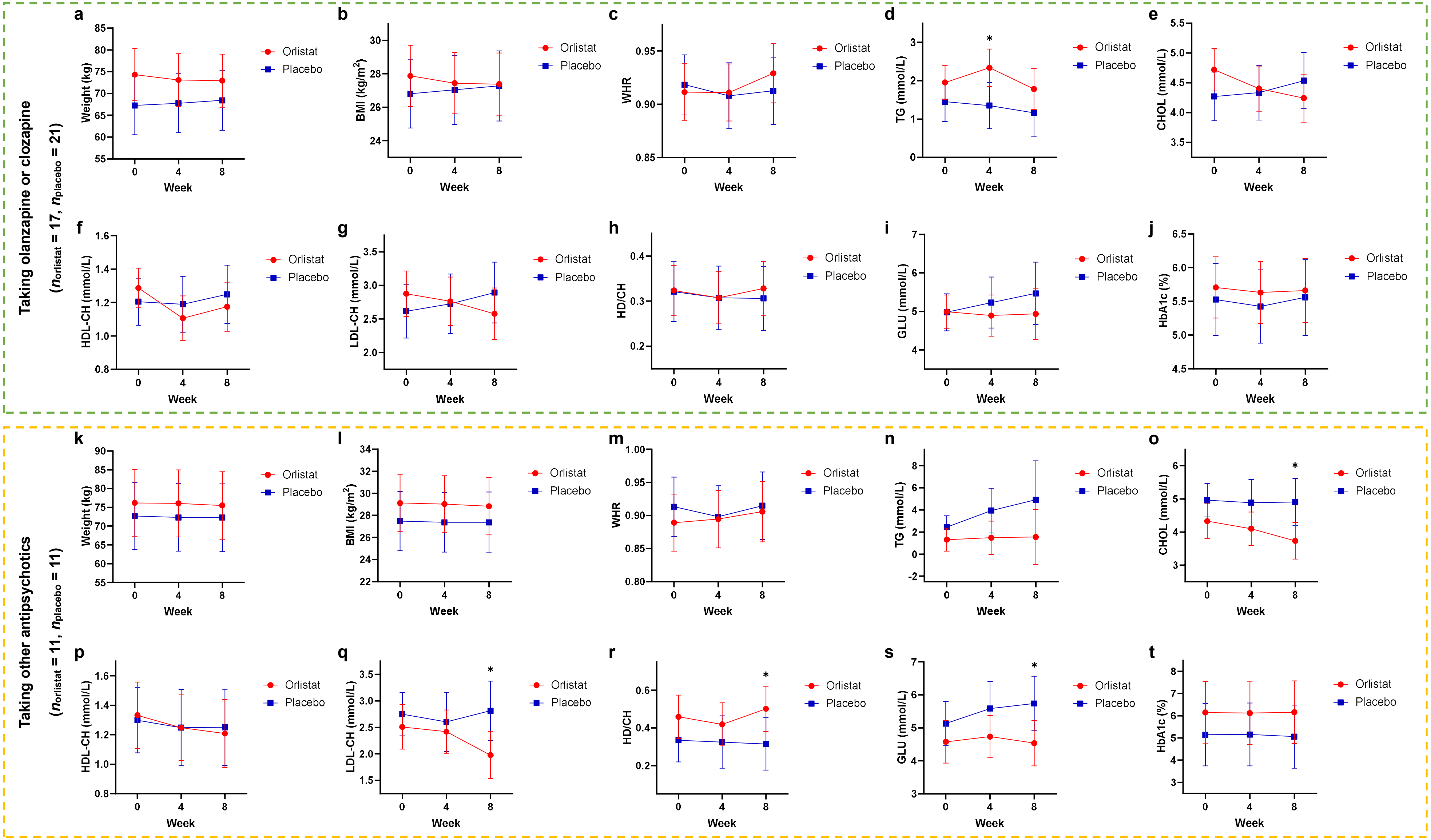

Figure S4. Mixed linear models: subgroup analyses by metabolic risk level of antipsychotics

Participants with or without olanzapine or clozapine treatment were analyzed separately using the intent-to-treat method. (a) to (j) show results for weight, BMI, WHR, TG, CHOL, HDL-CH, LDL-CH, HD/CH, GLU and HbA1c in patients taking olanzapine or clozapine. (k) to (t) show results for weight, BMI, WHR, TG, CHOL, HDL-CH, LDL-CH, HD/CH, GLU and HbA1c in patients taking other antipsychotics. The dots and error bars represent margins and 95% confidence intervals. ✱ Significant difference between groups (95% confidence interval for the contrast between margins does not include 0). Abbreviations: BMI: body mass index, WHR: waist-to-hip ratio, TG: triglyceride, CHOL: cholesterol, HDL-CH: high-density lipoprotein cholesterol, LDL-CH: low-density lipoprotein cholesterol, HD/CH: HDL-CH–to–CHOL ratio, GLU: glucose, HbA1c: glycosylated hemoglobin.

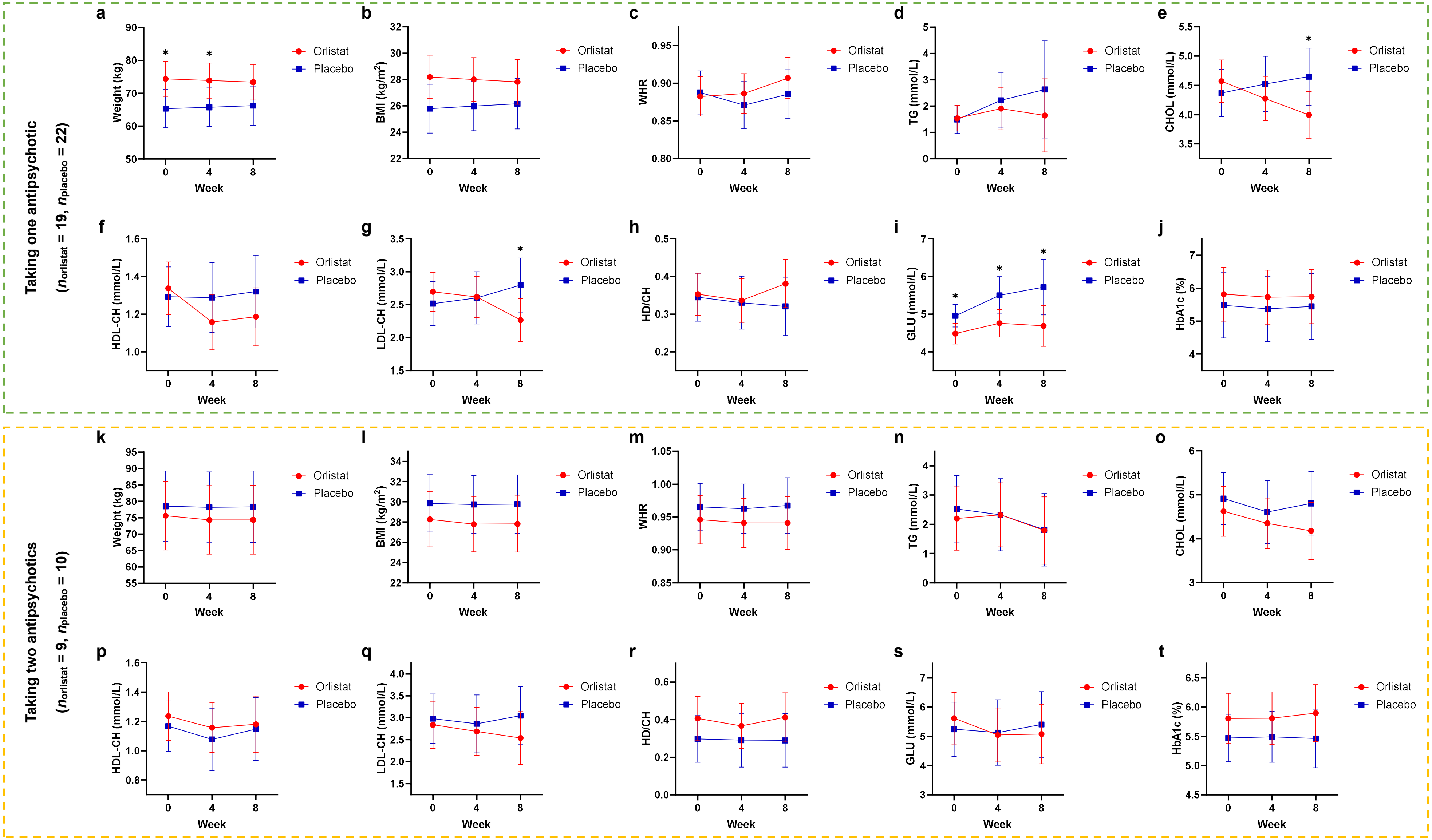

Figure S5. Mixed linear models: subgroup analyses by numbers of antipsychotics

Participants taking one or two antipsychotics were analyzed separately using the intent-to-treat method. (a) to (j) show results for weight, BMI, WHR, TG, CHOL, HDL-CH, LDL-CH, HD/CH, GLU and HbA1c in patients taking one antipsychotic. (k) to (t) show results for weight, BMI, WHR, TG, CHOL, HDL-CH, LDL-CH, HD/CH, GLU and HbA1c in patients taking two antipsychotics. The dots and error bars represent margins and 95% confidence intervals. ✱ Significant difference between groups (95% confidence interval for the contrast between margins does not include 0). Abbreviations: BMI: body mass index, WHR: waist-to-hip ratio, TG: triglyceride, CHOL: cholesterol, HDL-CH: high-density lipoprotein cholesterol, LDL-CH: low-density lipoprotein cholesterol, HD/CH: HDL-CH–to–CHOL ratio, GLU: glucose, HbA1c: glycosylated hemoglobin.

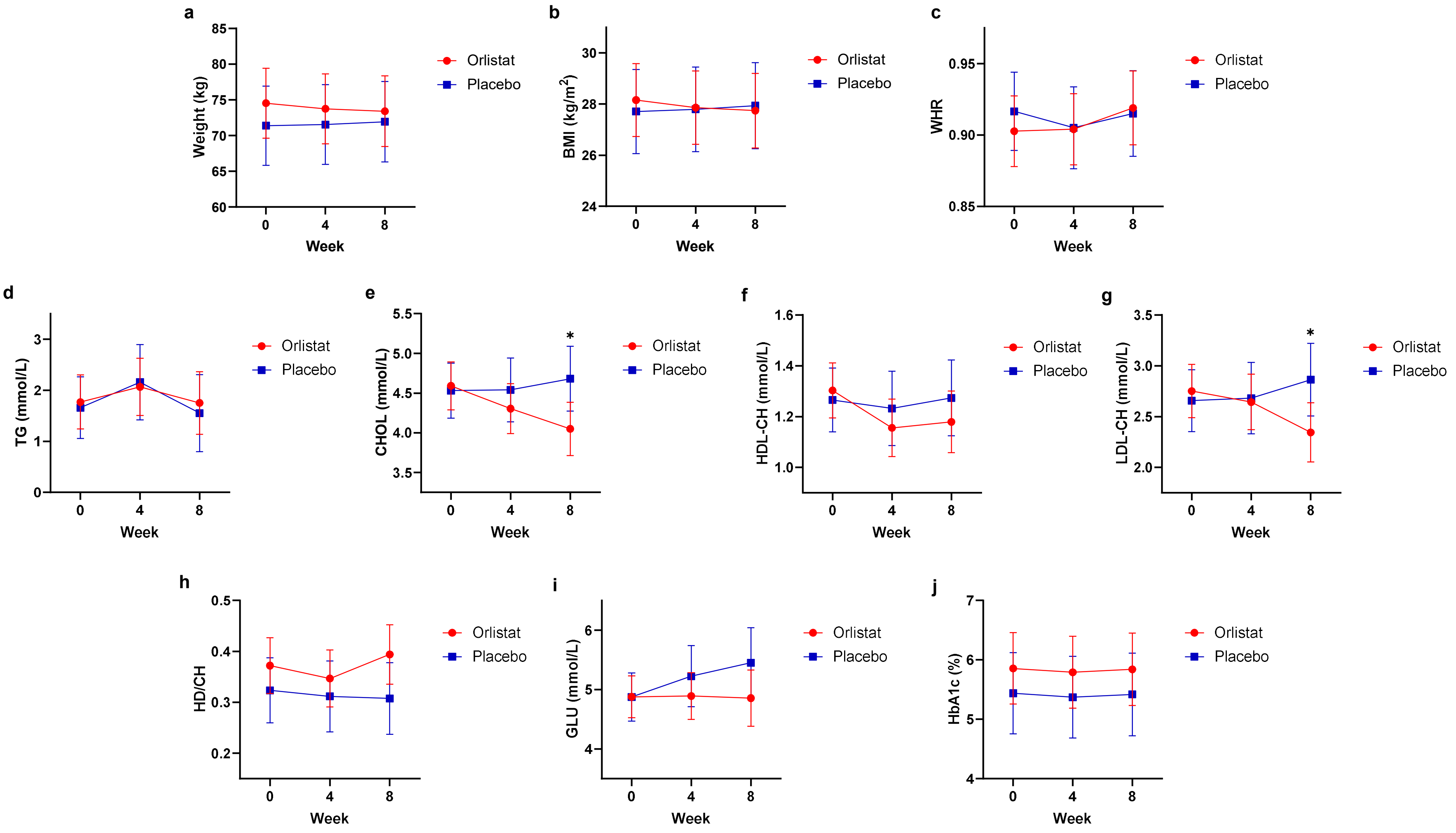

Figure S6. Mixed linear models for sensitivity analyses

Data were analyzed using the intent-to-treat method. Taking or not taking mood stabilizers was included as a covariant. (a) to (j) show results for weight, BMI, WHR, TG, CHOL, HDL-CH, LDL-CH, HD/CH, GLU and HbA1c, respectively. The dots and error bars represent margins and 95% confidence intervals. ✱ Significant difference between groups (95% confidence interval for the contrast between margins does not include 0). Abbreviations: BMI: body mass index, WHR: waist-to-hip ratio, TG: triglyceride, CHOL: cholesterol, HDL-CH: high-density lipoprotein cholesterol, LDL-CH: low-density lipoprotein cholesterol, HD/CH: HDL-CH–to–CHOL ratio, GLU: glucose, HbA1c: glycosylated hemoglobin.
